# CDKAL1-Related Single Nucleotide Polymorphisms Are Associated with Insulin Resistance in a Cross-Sectional Cohort of Greek Children

**DOI:** 10.1371/journal.pone.0093193

**Published:** 2014-04-02

**Authors:** Mathias Rask-Andersen, Gaëtan Philippot, George Moschonis, George Dedoussis, Yannis Manios, Claude Marcus, Robert Fredriksson, Helgi B. Schiöth

**Affiliations:** 1 Department of Neuroscience, Functional Pharmacology, Uppsala University, BMC, Uppsala, Sweden; 2 Department of Nutrition and Dietetics, Harokopio University, Athens, Greece; 3 Department for Clinical Science, Intervention and Technology, Karolinska Institutet, Division of Pediatrics, National Childhood Obesity Centre, Stockholm, Sweden; The University of Chicago, United States of America

## Abstract

Five novel loci recently found to be associated with body mass in two GWAS of East Asian populations were evaluated in two cohorts of Swedish and Greek children and adolescents. These loci are located within, or in the proximity of: CDKAL1, PCSK1, GP2, PAX6 and KLF9. No association with body mass has previously been reported for these loci in GWAS performed on European populations. The single nucleotide polymorphisms (SNPs) with the strongest association at each loci in the East Asian GWAS were genotyped in two cohorts, one obesity case control cohort of Swedish children and adolescents consisting of 496 cases and 520 controls and one cross-sectional cohort of 2293 nine-to-thirteen year old Greek children and adolescents. SNPs were surveyed for association with body mass and other phenotypic traits commonly associated with obesity, including adipose tissue distribution, insulin resistance and daily caloric intake. No association with body mass was found in either cohort. However, among the Greek children, association with insulin resistance could be observed for the two CDKAL1-related SNPs: rs9356744 (*β* = 0.018, p = 0.014) and rs2206734 (*β* = 0.024, p = 0.001). CDKAL1-related variants have previously been associated with type 2 diabetes and insulin response. This study reports association of CDKAL1-related SNPs with insulin resistance, a clinical marker related to type 2 diabetes in a cross-sectional cohort of Greek children and adolescents of European descent.

## Introduction

The worldwide obesity epidemic is a major public health concern associated with increased morbidity and mortality [1]. Obesity shows comorbidity with e.g. type 2 diabetes mellitus (T2DM), cardiovascular disease, hypertension and certain types of cancer [2]–[4]. However, the exact mechanisms behind the development of obesity are not fully understood. Even though recent lifestyle changes have likely triggered the increased prevalence of obesity, a genetic predisposition has been indicated to substantially contribute to the etiology of this condition [5]–[7]. As of today, at least 32 obesity-associated loci have been identified in by the GIANT-consortium [8]–[9]. These findings come from meta-analyses of genome-wide association studies (GWAS) and are predominantly based on subjects of European ancestry.

Recently, two GWAS identified five novel loci associated with BMI in East Asians [10]–[11] that have, as of yet, not been associated with body mass in European cohorts. The loci were located in or near the CDKAL1, PCSK1, GP2, PAX6 and KLF9 genes. The aim of this study was to determine if these SNPs were associated with BMI also in Europeans. We therefore analyzed the strongest associated SNPs reported in the two GWAS performed on East Asian populations: rs9356744, rs2206734, rs652722, rs11142387, rs261967 and rs12597579 for associations with obesity and body mass in a case control cohort for obesity consisting of 1016 Swedish children and adolescents recruited in the Stockholm area, as well as in a cross-sectional national cohort of 2293 Greek children and adolescents recruited from schools. Phenotypic traits commonly associated with BMI were also surveyed, including adipose tissue distribution (hip- and waist circumference and thickness of skinfolds), homeostasis model assessment of insulin resistance (HOMA-IR) and daily caloric intake [12].

## Methods

### Ethics statement: Healthy Growth Study, Greek children and adolescents

An extended letter and a consent form were provided to each parent having a child in one of the primary schools explaining the aims of the study. Parents who agreed to participate in the study had to sign the consent form and provide their contact details. The study was approved by the Greek Ministry of Education and the Ethical Committee of Harokopio, University of Athens, Athens, Greece.

### Ethics statement: Case control study for obesity, Swedish children and adolescents

The study was approved by the Regional Committee of Ethics, Stockholm. All subjects, or their legal guardians, gave their written informed consent.

### Healthy Growth Study, Greek children and adolescents

The Healthy Growth Study, a large scale cross-sectional epidemiological study, was initiated in May 2007. The survey population consisted of 2657 school children aged 9–13 years attending the fifth and sixth grade of primary school (Table 1), as previously described [13]–[15]. Standard procedures and equipment were used to measure body weight and height in all study participants. Body weight without shoes in the minimal amount of clothing possible was measured using a Seca digital scale Model 770 (Seca Alpha, Hamburg, Germany), to the nearest 10 g. Height was measured to the nearest 0.1 cm, in standing position without shoes, shoulders in a relaxed position, arms hanging freely and head aligned in Frankfurt plane, using a commercial stadiometer (Leicester Height Measure, Invicta Plastics Ltd, Oadby, UK). BMI z-score was calculated relative to the International Obesity Task Force (IOTF) definitions [16]. Blood samples were obtained for screening tests after a 12 h overnight fast. Plasma separation of blood was performed by centrifugation (3000 rpm for 15 min). Serum insulin concentrations were measured using a chemiluminescence immunoassay (Kyowa Medex Ltd, Minami-Ishiki, Japan). Insulin resistance (IR) was calculated through the homeostasis model assessment (Equation 1) [17]. 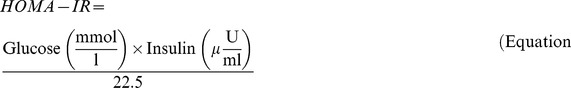
(Equation\;1)


**Table 1:** Descriptive characteristics of the cohorts of Greek and Swedish children and adolescents.

	N (boys/girl)	Age (years)	Body weight (kg)	Length (m)	BMI z-score	HOMA-IR	Daily caloric intake (kcal/day)
*Greek cohort*							
	2293 (1154/1132)	11.2±0.7	45.3±11.1	1.49±7.8	0.84±1.27	2.381±1.720	1785±552.4
*Swedish cohort*							
Controls	520 (253/267)	17.0±0.9	63.5±10.1	1.73±0.09	−3.0×10^−4^±0.8		
Cases	496 (236/260)	12.8±3.2	92.9±29.1	1.59±0.16	3.35±1.65		

DNA for genotyping was available for 2293 individuals (1154 males and 1132 females). Waist and hip circumference was measured at standing position using non-elastic tape (Hoechstmass, Sulzback, Germany) to the nearest 0.1 cm. Measurements were taken around the trunk, at the umbilicus midway between the lower rib margin and the iliac crest. Thickness of four skinfolds (triceps, biceps, subscapular and suprailiac) were measured to the nearest 0.1 mm with a Large skinfold caliper (Cambridge, Maryland). Triceps and biceps skinfold thickness was measured on the right arm hanging freely at the side of the body, with the skinfold being picked up 1 cm below the midpoint mark over the triceps and biceps. Subscapular skinfold thickness was measured in standing position, arms hanging in a relaxed position after identifying the inferior angle of the scapula. The skinfold was picked up 1 cm below the subscapular mark. Suprailiac skinfold thickness was measured above the iliac crest along the axis of the anterior line. Pubertal maturation (Tanner Stage) was determined by four well-trained pediatricians. Breast development in girls and genital development in boys was examined according to the pubertal maturation classification (Tanner Stage 1 to 5) [18]. The study was approved by the Greek Ministry of Education and the Ethical Committee of Harokopio University, Athens, Greece.

### Case control study for obesity, Swedish children and adolescents

The cohort of Swedish children was comprised of 1016 children and adolescents in two groups, as described previously [13], [15]. One group consisted of 496 obese children (236 boys and 260 girls) registered at the National Childhood Obesity centre at Karolinska University Hospital, Huddinge, Sweden. The second group consisted of 520 healthy adolescents with normal weight (253 boys and 267 girls) recruited from 17 upper secondary schools in the Stockholm area, Sweden (Table 1). Body weight and height were measured to the nearest 0.1 kg and 1 cm, respectively. BMI z-score was calculated according to the International Obesity Task Force (IOTF) definitions [16]. A BMI z-score >2 is commonly utilized as a cutoff for defining obesity. In the obese group, patients with T2DM were excluded. Subjects in the control group that were overweight, obese or had metabolic diseases were also excluded.

### SNP genotyping

Genotyping of gene variants was carried out using the pre-designed Taqman single-nucleotide polymorphism genotyping assay (Applied Biosystems, Foster city, USA) and an ABI7900 genetic analyzer with SDS 2.2 software at the Uppsala Genome Center (http://www.genpat.uu.se/node462).

### Statistical analysis

All statistical analyses were made using PLINK (http://pngu.mgh.harvard.edu/purcell/plink) [19]. Quantitative skewed variables were log transformed if needed to meet the assumptions of parametric statistics. Deviation from Hardy-Weinberg equilibrium was tested for using the Pearson's χ^2^- test. Association between genotypes and phenotypes in the Greek cohort were analyzed with linear regression, assuming an additive model. All analyses in the Greek cohort were adjusted for age, gender and pubertal development (Tanner stage). Analyses of secondary phenotypic traits were also adjusted for BMI z-score. The Swedish cohort was analyzed using logistic regression and was adjusted for age and gender. Results are presented as odds ratios (OR) with 95% confidence interval (CI). We applied the False Discovery Rate by Benjamini and Hochberg [20] to correct for multiple testing. Associations were considered significant if the adjusted p value <0.05.

### Power calculation

Power calculation for the Swedish and Greek cohorts were performed in CaTS power calculator (http://www.sph.umich.edu/csg/abecasis/CaTS/) and Quanto (http://hydra.usc.edu/gxe/) utilizing effect sizes reported in the previous GWAS [10]–[11] as well as child and adolescent obesity prevalence of 4.3% [21]. We estimate a 20–40% power, depending on effect size and allele frequency, to observe effects on BMI for the studied SNPs of medium to high allele frequency (CDKAL1-, KLF9-, PCSK1- and PAX9-related SNPs) in the cohort of Greek children and adolescents. Our power was lower (∼10%) for detecting association of the GP2-related SNP rs12597579 with body mass due to the low frequency of this SNP (7%). In the case control cohort of Swedish obese children and adolescents, we estimate a 40–70% power to detect associations of the medium to high frequency SNPs with obesity, depending on allele frequency. Again, the low frequency of rs12597579 leads to a lower power for this SNP at about 18%.

## Results

Genotyping was performed on 2293 subjects in the Greek cohort and 1016 subjects in the Swedish cohort. The call rate was between 98.5–99.7%. No deviation from Hardy-Weinberg equilibrium was observed. In our analysis we were unable to observe any effect of the studied SNPs on body mass in the cohort of Greek children and adolescents (Table S1). We were also unable to observe any association with obesity in the case control study of Swedish children and adolescents (Table S2). Analyses of waist-to-hip ratio and skinfold thickness also failed to reveal any association with the studied SNPs (Table S3).

### Association of CDKAL1-related SNPs rs9356744 and rs2206734 with insulin resistance

Linear regression revealed associations between HOMA-IR and two SNPs within the CDKAL1 gene: rs9356744 and rs2206734 (β = 0.018, adjusted p = 0.042 and β = 0.024, adjusted p = 0.025 respectively) in models adjusted for age, gender, body mass and pubertal development (Table 2).

**Table 2:** Association of SNPs with HOMA-IR in the Greek cohort.

						HOMA-IR		
SNP	Gene	genotypic distribution	HWE	MAF (%)	*n*	*β*	*p*-value	FDR
rs261967	PCSK1	CC/CA/AA (444/1083/749)	0.15	43.3	1973	0.0027	0.69	0.99
rs9356744	CDKAL1	CC/CT/TT (229/997/1054)	0.81	31.9	1977	0.018	0.014^*^	0.042*
rs2206734	CDKAL1	TT/TC/CC (114/773/1393)	0.62	22.0	1976	0.024	0.0041^**^	0.025*
rs11142387	KLF9	AA/AC/CC (470/1174/636)	0.10	46.4	1976	−0.10	0.99	0.99
rs652722	PAX6	TT/TC/CC (157/858/1265)	0.48	25.7	1976	0.0012	0.88	0.99
rs12597579	GP2	TT/TC/CC (13/291/1973)	0.52	7.00	1973	−0.024	0.08	0.16

*β* - regression coefficient. MAF – minor allele frequency. HWE – Hardy Weinberg equilibrium deviation test presented as p-value. *p<0.05, **p<0.005.

Linear regression was used to analyze association with phenotypic traits. Models were adjusted for BMI z-score, gender, age and pubertal development (tanner stage).

### Association of rs652722 with daily caloric intake

Linear regression revealed association between rs652722 and daily caloric intake among the Greek children. The minor allele of rs652722, near the PAX6 gene was associated with lower daily caloric intake (β = −0.010, nominal p = 0.020) in models adjusted for age, gender, body mass and pubertal development. However, this association was not significant after correcting for multiple testing (Table 3).

**Table 3:** Analysis of association of rs652722 with daily caloric intake in the Greek cohort.

					Daily caloric intake		
SNP	Gene	Genotypic distribution	MAF (%)	n	*β*	*p* -value	*FDR*
rs261967	PCSK1	CC/CA/AA (229/997/1054)	43.3	2250	−0.0017	0.65	0.76
rs9356744	CDKAL1	CC/CT/TT (229/997/1054)	31.9	2254	0.0044	0.29	0.58
rs2206734	CDKAL1	TT/TC/CC (114/773/1393)	22.0	2254	0.0060	0.19	0.57
rs11142387	KLF9	AA/AC/CC (470/1174/636)	46.4	2254	−0.0025	0.53	0.76
rs652722	PAX6	TT/TC/CC (157/858/1265)	25.7	2254	−0.010	0.02*	0.12
rs12597579	GP2	TT/TC/CC (13/291/1973)	7.00	2251	−0.0023	0.76	0.76

*β* - regression coefficient. MAF – minor allele frequency. HWE – Hardy Weinberg equilibrium deviation test presented as p-value. *p<0.05.

Linear regression was used to analyze association with phenotypic traits. Models were adjusted for BMI z-score, gender, age and pubertal development (tanner stage).

## Discussion

The strongest associated SNPs at five loci associated with BMI in East Asians [10]–[11] were evaluated for association with BMI and related traits in two cohorts of children of European ancestry: one cross-sectional cohort of Greek children and one case-control study of obese Swedish children and adolescents. The effects on body mass observed in East Asian populations could not be replicated in our studies indicating heterogenic effects of these loci across European and East Asian populations. These results are in line with reports from the GIANT consortium [22]. Despite reporting some directionally consistent effects as the East Asian GWAS, the effects of variants at these loci were not powerful enough to reach the criteria for statistical significance in the analysis by the GIANT consortium. It must be highlighted that our study utilized cohorts of children, which could indicate that genetic effects of variations at these loci have a higher penetrance in adults, as the two GWAS on East Asians primarily performed their studies on adult populations [10]–[11]. Statistical power is also a potential limiting factor due to the relatively small sizes of our cohorts. In the case of the GP2-related SNP rs12597579, this may be further compounded by the low frequency of its minor allele (minor allele frequency approximately 5–7%).

However, the SNPs within the T2DM associated gene CDKAL1, rs9356744 and rs2206734, were observed to be associated with HOMA-IR (*β* = 0.02, adjusted p = 0.042 and *β* = 0.025, adjusted p = 0.025, respectively) in linear regression models co-varied for body mass, age, gender and pubertal development (Table 2). HOMA-IR is an estimate of IR which describes the interplay between plasma glucose and insulin release from the pancreatic islets. It is determined through measurements of fasting insulin and plasma glucose [17], [23]. An elevated IR, a cut-off value 2.60 is commonly used, denotes an insufficiency of insulin-mediated glucose uptake and is one of the central features of T2DM and also one of the key features of the metabolic syndrome [24]–[25]. A study in U.S subjects found obese children and adolescents (12–19 years old) to have a higher IR compared to normal weight children and adolescents (4.93 vs. 2.30) [26]. Insulin resistance has also been suggested as a prognostic marker for development of T2DM although causality has not been firmly established [27]–[28].

The directionalities of the effects of rs2206734 and rs9356744 on BMI observed in East Asians [10]–[11] are opposite compared to the effects on insulin resistance observed by us. However, our findings are in line with those of Okada et al. who also reported the T allele of rs2206734, which was associated with lower BMI in a cohort of East Asians, to be associated with an increased risk of T2DM [10]. Our findings are also in line with several studies showing associations of CDKAL1-related genetic variants with T2DM [29]–[30]. CDKAL1 encodes a methylthiotransferase that modifies tRNA to enhance the translational accuracy of the pro-insulin transcript [31]. A study with knockout mice indicated the involvement of CDKAL1 in exocytosis of first phase insulin in β-cells [32].

A trend towards associations with body mass and IR were observed for the GP2-related SNP rs12597579 (*β* = −0.024, nominal p = 0.079). The low minor allele frequency of this SNP unfortunately limits the statistical power of our analysis. Replication in larger cohorts may clarify potential effects of this locus on body mass and IR. GP2 is an interesting candidate gene at this locus due to its high expression in the pancreas. Rs12597579 is located ∼65 kb downstream from the GP2 gene on chromosome 16. GP2 is highly expressed in the pancreas and especially within the islets of Langerhans [33]–[34]. Specifically, the GP2 protein is expressed in the secretory granules of acinar-cells and is the most abundant protein in the pancreatic secretory granule membrane, accounting for 35% of total membrane protein [35]–[36]. More recently, GP2 has been shown to be present in the microfold (M) cells of the follicle-associated epithelium (FAE) of intestinal Peyer's patches [37]. There is evidence of a potential autoantigen function of GP2 for pancreatic autoantibody (PAB), which is involved in Crohn's disease [38], but the full function of the GP2 gene is yet to be elucidated. Due to the high, close to exclusive, expression of GP2 in the islets of Langerhans, according to the gene atlas of the mouse and human protein encoding transcripts [39], accessed via BioGPS.org, evaluation of genetic variants in or near this gene could be highly relevant to diabetes research.

Some indication of association of rs652722 with a lower caloric intake was observed at nominal p-values, but not when correcting for multiple testing. According to the SCAN database [40] this SNP is in linkage disequilibrium with several other SNPs that influence genes potentially important for body weight regulation [11]. The PAX6 gene is located in the closest proximity of this locus and is expressed in all endocrine cells, e.g. ghrelin cells, during development [41]. After development, the expression of PAX6 is necessary in the control of, among several other hormones, glucagon and insulin [42]–[43]. Homozygous PAX6 deleted mice showed symptoms of diabetes and severe weight loss [44], which points to a possible connection between PAX6-associated SNPs and metabolic function.

In summary, we observe associations between SNPs identified to be associated to body mass in two recently published GWAS performed in East Asian subjects with phenotypic traits associated with T2DM, in two cohorts of children and adolescents of European descent. In a cohort of Greek children and adolescents, CDKAL1-related SNPs rs9356744 and rs9356734 were observed to be associated with IR. The results provide candidate genetic markers for IR, which may be of great importance in both research and clinical practice.

## Supporting Information

Table S1 **Association of SNPs with BMI z-score in the Greek cohort.** Linear regression was used to analyze association with BMI z-score. Models were adjusted for gender, age and pubertal development (tanner stage).
(DOCX)

Table S2 **Logistic regression was used to analyze association of SNPs with obesity in the cohort of Swedish children and adolescents.** Models were adjusted for gender and age.
(DOCX)

Table S3 **Association of SNPs with adipose tissue distributions in the Greek cohort.** Linear regression was used to analyze association with phenotypic traits. Models were adjusted for BMI z-score, gender, age and pubertal development (tanner stage).
(DOCX)
