# Supplementary material for: CDKAL1-Related Single Nucleotide Polymorphisms Are Associated with Insulin Resistance in a Cross-Sectional Cohort of Greek Children
Source: PLoS One. 2014 Apr 2;9(4):e93193. doi: 10.1371/journal.pone.0093193 (PMC3973700; doi:10.1371/journal.pone.0093193)
Supplement: Table S1 — Association of SNPs with BMI z-score in the Greek cohort. Linear regression was used to analyze association with BMI z-score. Models were adjusted for gender, age and pubertal development (tanner stage). (DOCX) [file pone.0093193.s001.docx]

Table S1. Association of SNPs with BMI z-score in the Greek cohort. Linear regression was used to analyze association with BMI z-score. Models were adjusted for gender, age and pubertal development (tanner stage).

| **SNP** | **genotypic distribution** | **HWE** | **MAF (%)** | **BMI z-score** | | |
| --- | --- | --- | --- | --- | --- | --- |
|  |  |  |  | ***n*** | ***β*** | ***p*-value** |
| rs261967 | CC/CA/AA  444/1083/749 | 0.15 | 43.3 | 2268 | -0.023 | 0.51 |
| rs9356744 | CC/CT/TT  229/997/1054 | 0.81 | 31.9 | 2272 | 0.018 | 0.64 |
| rs2206734 | TT/TC/CC  114/773/1393 | 0.62 | 22.0 | 2272 | 0.019 | 0.66 |
| rs11142387 | AA/AC/CC  470/1174/636 | 0.10 | 46.4 | 2272 | -0.020 | 0.58 |
| rs652722 | TT/TC/CC  157/858/1265 | 0.48 | 25.7 | 2272 | 0.037 | 0.36 |
| rs12597579 | TT/TC/CC  13/291/1973 | 0.52 | 7.00 | 2269 | -0.13 | 0.061 |

*β* - regression coefficient. MAF – minor allele frequency. HWE – Hardy Weinberg equilibrium deviation test presented as p-value.
