# Supplementary material for: CDKAL1-Related Single Nucleotide Polymorphisms Are Associated with Insulin Resistance in a Cross-Sectional Cohort of Greek Children
Source: PLoS One. 2014 Apr 2;9(4):e93193. doi: 10.1371/journal.pone.0093193 (PMC3973700; doi:10.1371/journal.pone.0093193)
Supplement: Table S2 — Logistic regression was used to analyze association of SNPs with obesity in the cohort of Swedish children and adolescents. Models were adjusted for gender and age. (DOCX) [file pone.0093193.s002.docx]

Table S2. Logistic regression was used to analyze association of SNPs with obesity in the cohort of Swedish children and adolescents. Models were adjusted for gender and age.

| **SNP** | **Genotypic distribution** | **HWE** | **MAF (%)** | **Odds ratio (95% CI)** | ***p*-value** |
| --- | --- | --- | --- | --- | --- |
| rs261967 | CC/CA/AA  216/483/301 | 0.41 | 45.75 | 1.02 (0.78-1.30) | 0.89 |
| rs9356744 | CC/CT/TT  106/418/475 | 0.34 | 31.53 | 1.12 (0.83-1.50) | 0.47 |
| rs2206734 | TT/TC/CC  35/294/672 | 0.67 | 18.18 | 1.23 (0.87-1.74) | 0.25 |
| rs11142387 | AA/AC/CC  235/483/285 | 0.28 | 47.51 | 0.94 (0.72-1.23) | 0.65 |
| rs652722 | TT/TC/CC  71/416/514 | 0.31 | 27.87 | 0.92 (0.67-1.26) | 0.60 |
| rs12597579 | TT/TC/CC  4/95/901 | 0.33 | 5.15 | 0.82 (0.43-1.59) | 0.56 |

*β* - regression coefficient. MAF – minor allele frequency. HWE – Hardy Weinberg equilibrium deviation test presented as p-value.
