# Supplementary material for: CDKAL1-Related Single Nucleotide Polymorphisms Are Associated with Insulin Resistance in a Cross-Sectional Cohort of Greek Children
Source: PLoS One. 2014 Apr 2;9(4):e93193. doi: 10.1371/journal.pone.0093193 (PMC3973700; doi:10.1371/journal.pone.0093193)
Supplement: Table S3 — Association of SNPs with adipose tissue distributions in the Greek cohort. Linear regression was used to analyze association with phenotypic traits. Models were adjusted for BMI z-score, gender, age and pubertal development (tanner stage). (DOCX) [file pone.0093193.s003.docx]

Table S3. Association of SNPs with adipose tissue distributions in the Greek cohort. Linear regression was used to analyze association with phenotypic traits. Models were adjusted for BMI z-score, gender, age and pubertal development (tanner stage).

| **SNP** | **genotypic distribution** | **MAF (%)** | **n** | **Waist to hip ratio** | | **n** | **Bicep skinfold thickness** | | **Tricep skinfold thickness** | | **Suprilliac skinfold** | | **Subscapular skinfold** | |
| --- | --- | --- | --- | --- | --- | --- | --- | --- | --- | --- | --- | --- | --- | --- |
|  |  |  |  | ***β*** | **p** |  | ***β*** | **p** | ***β*** | **p** | ***β*** | **p** | ***β*** | **p** |
| rs261967 | CC/CA/AA  444/1083/749 | 43.3 | 2269 | 0.0013 | 0.40 | 2270 | -0.042 | 0.67 | -0.047 | 0.70 | -0.0013 | 0.77 | -0.00092 | 0.81 |
| rs9356744 | CC/CT/TT  229/997/1054 | 31.9 | 2273 | -0.00015 | 0.93 | 2274 | -0.044 | 0.68 | -0.041 | 0.75 | 0.0012 | 0.80 | -0.0015 | 0.70 |
| rs2206734 | TT/TC/CC  114/773/1393 | 22.0 | 2273 | 0.00096 | 0.62 | 2274 | -0.035 | 0.77 | 0.036 | 0.81 | 0.0051 | 0.34 | 0.00078 | 0.86 |
| rs11142387 | AA/AC/CC  470/1174/636 | 46.4 | 2273 | 0.0012 | 0.44 | 2274 | 0.050 | 0.62 | 0.091 | 0.46 | 0.0028 | 0.53 | 0.0024 | 0.53 |
| rs652722 | TT/TC/CC  157/858/1265 | 25.7 | 2273 | -0.0014 | 0.42 | 2274 | -0.017 | 0.88 | -0.094 | 0.49 | -0.0042 | 0.40 | -0.00062 | 0.88 |
| rs12597579 | TT/TC/CC  13/291/1973 | 7.00 | 2270 | -0.0013 | 0.65 | 2271 | 0.059 | 0.76 | 0.16 | 0.49 | -0.0023 | 0.79 | -0.0011 | 0.88 |

*β* - regression coefficient. MAF – minor allele frequency.
